# Comparative Feeding and Defecation Behaviors of *Trypanosoma cruzi*-Infected and Uninfected Triatomines (Hemiptera: Reduviidae) from the Americas

**DOI:** 10.3390/insects16020188

**Published:** 2025-02-10

**Authors:** Keswick C. Killets, Jillian Wormington, Italo Zecca, Luis Fernando Chaves, Gabriel L. Hamer, Sarah A. Hamer

**Affiliations:** 1 College of Veterinary Medicine and Biomedical Sciences, Texas A&M University, College Station, TX 77843, USA; kkillets@cvm.tamu.edu (K.C.K.); jillianwormington@gmail.com (J.W.); italobzecca@gmail.com (I.Z.); 2 Department of Life Sciences, Wayne State College, Wayne, NE 68787, USA; 3 Instituto Conmemorativo Gorgas de Estudios de la Salud, Ciudad de Panama Apartado Postal 0816-02593, Panama; lfchavs@gmail.com; 4 Department of Environmental and Occupational Health, School of Public Health and Department of Geography, Indiana University, Bloomington, IN 47405, USA; 5 Department of Entomology, Texas A&M University, College Station, TX 77843, USA; ghamer@tamu.edu

**Keywords:** feeding and defecation behaviors, triatomines, *Trypanosoma cruzi*

## Abstract

Triatomines are vectors of *Trypanosoma cruzi* (Kinetoplastida: Trypanosomatidae), the agent of Chagas disease. Stercorarian transmission occurs when infectious parasites are passed in insect feces to vertebrates through the biting wound or mucosa. Defecating on hosts during or shortly after blood feeding is, therefore, critical for transmission, and delayed triatomine defecation behavior has been posited to contribute to a low incidence of human Chagas disease in the U.S. We allowed nymphal *T. cruzi*-infected and uninfected *Triatoma gerstaeckeri* (Stål, 1859) and *Triatoma sanguisuga* (LeConte, 1855) to interact with restrained guinea pigs and measured insect feeding and defecation events; South American *Rhodnius prolixus* (Stål, 1859; Latin America) served as a comparison group. In 148 trials, 40.0% of insects fed, of which 71.2% defecated. Compared to *R. prolixus*, *T. gerstaeckeri* had >9 times higher odds of feeding, and *T. sanguisuga* fed longer. Observations of defecation while feeding occurred across all three species. The post-feeding defecation interval (PFDI) of *R. prolixus* was significantly shorter (4.54 ± 2.46 min) than that of *T. gerstaeckeri* (9.75 ± 2.52 min) and *T. sanguisuga* (20.69 ± 8.98 min). Furthermore, the PFDI was shorter for TcI-infected insects compared to uninfected insects. *Triatoma gerstaeckeri* and *T. sanguisuga* are capable of stercorarian transmission, although the calculated metrics suggest they are less efficient vectors than *R. prolixus*.

## Simple Summary

Triatomines, also known as kissing bugs, are insect vectors of *Trypanosoma cruzi*, the agent of Chagas disease. Transmission occurs when infectious parasites are passed in insect feces to vertebrates through the biting wound or mucosa. Defecating on hosts during or shortly after blood feeding is, therefore, critical for transmission, and delayed triatomine defecation behavior has been posited to contribute to a low incidence of human Chagas disease in the United States. We allowed immature *T. cruzi*-infected and uninfected *Triatoma gerstaeckeri* and *Triatoma sanguisuga*, both species of vectors found in the United States, to interact with restrained guinea pigs and measured insect feeding and defecation events. We use a South American species, *Rhodnius prolixus*, as a comparison group. Results showed that *T. gerstaeckeri* had >9 times higher odds of feeding, and *T. sanguisuga* fed longer compared to *R. prolixus*. Observations of defecation while feeding occurred across all three species. The post-feeding defecation interval of *R. prolixus* was significantly shorter than that of *T. gerstaeckeri* and *T. sanguisuga*. The post-feeding defecation interval was shorter for TcI-infected insects than uninfected individuals. *Triatoma gerstaeckeri* and *T. sanguisuga* are capable of transmission, although the calculated metrics suggest they are less efficient vectors than *R. prolixus*.

## 1. Introduction

Triatomines (Hemiptera: Reduviidae) transmit *Trypanosoma cruzi*, the etiological agent of Chagas disease, and require bloodmeals to develop throughout their hemimetabolous life cycles. Over 150 triatomine species distributed from the Southern U.S. to Argentina and Chile are potential vectors of *T. cruzi* [1,2]. Triatomines exhibit stercorarian biological transmission, in which they transmit the infectious state of *T. cruzi* via fecal contamination to the host. If an infected triatomine feeds on a host and defecates at the same time or shortly after, the probability of host infection with the infectious parasite through the bite wound or mucosa increases [3]. Accordingly, there is a long-standing interest in triatomine defecation behavior as it relates to the risk of *T. cruzi* vectorial transmission [4,5,6,7,8,9,10,11,12,13].

Vectorial capacity (VC) is a concept that allows scientists to estimate vector transmission potential by incorporating all the intrinsic and extrinsic factors that regulate this process [14]. VC includes factors such as vector competence, extrinsic incubation period, the vector density in relation to humans, daily survival rate, and probability of the vector feeding on the host [14]. For triatomines and the transmission of *T. cruzi*, intrinsic factors include the ability of triatomines to ingest and then transmit *T. cruzi* (vector competence), the infectious dosage of parasites in triatomine feces [15], the host utilization of triatomines and contact with different vertebrates such as dogs or humans [16], and the frequency of triatomine colonization of domicile environments [17]. Feeding and defecation behaviors are a critical part of vector competence as they relate to the probability of infectious stages of *T. cruzi* in feces coming into contact and infecting a host. Beyond VC, additional transmission routes of *T. cruzi* further complicate this pathosystem and include oral consumption of infected triatomines or feces by wild hosts, dogs, and other mammals through ingestion or grooming, which is an efficient route of *T. cruzi* [18,19], or by consumption of contaminated foods by animals and humans [20].

Studies of feeding and defecation behaviors of triatomines have been conducted for decades. It is assumed that South American triatomines are more efficient vectors of *T. cruzi* because they generally have shorter post-feeding defecation intervals (PFDIs) [4,12,21,22,23], as compared to North American species [4,6,7,9,12]. Although not all South American triatomine species have short PFDIs, studies have suggested that other domiciliary species with longer PFDIs could serve as secondary vectors of Chagas disease [24]. The feeding and defecation behaviors of North American species may be the main contributor to the lower human burden of Chagas disease in the U.S. relative to Latin America [6,7,9,25,26,27], as autochthonous cases in the U.S. are rare [28], despite established vector populations in both locations. However, only selected U.S. taxa have been examined, with little attention to how infection with *T. cruzi* may alter defecation behaviors [6,7,9,12]. Additional factors that may contribute to fewer autochthonous human cases in the U.S. include (1) low testing rates and underreporting of Chagas disease cases, (2) generally robust housing to prevent domestication of triatomines, (3) the scarcity of triatomine domicile colonization [29], and (4) variation in the pathogenicity of the parasite.

*Triatoma gerstaeckeri* (Stål) is the most commonly collected species in Texas [30,31] and is distributed throughout most of Texas, parts of New Mexico, and it has been documented in 10 states in northern Mexico [32]. *Triatoma sanguisuga* (LeConte), known as the ‘eastern blood-sucking conenose bug’, is broadly distributed from Texas to the east coast of the U.S. [31]. *Triatoma gerstaeckeri* and *T. sanguisuga* are considered the two most epidemiologically important vector species in Texas, and they both have relatively high *T. cruzi* infection prevalence [33] (45–70% for *T. gerstaeckeri* [12,30,33,34]; 25–67% for *T. sanguisuga* [12,30,34,35]) and are frequently encountered in or around households in Texas [33]. In the United States, *T. cruzi* discrete typing units (DTUs) TcI and TcIV are the most predominant, with TcI being associated with human infections, though other DTUs have been found in autochthonous human cases in the US [29,36]. Additionally, there is evidence that the TcIV strain found in the United States is distinct to its South American counterpart phylogenetically [37]. In a comprehensive study, *Triatoma gerstaeckeri* is more likely to carry DTU TcI, while *T. sanguisuga* is more likely to carry DTU TcIV [33].

*Rhodnius prolixus* (Stål) is native to northern South America, and spread to Central America; it is a competent vector for *T. cruzi* and is highly domiciliated [38,39,40]. It is the most common laboratory triatomine model [41,42]. *Rhodnius prolixus*, and other South American species [21] have been shown to have higher vectorial capacity given short PFDIs compared to triatomine species found in the U.S. [7,12]. Thus, *R. prolixus* is a good model for the comparison of feeding and defecation behaviors with the U.S. species.

The objective for this study was to examine feeding and defecation behaviors of *T. cruzi*-infected and uninfected *T. gerstaeckeri* and *T. sanguisuga* in the presence of live hosts in comparison with colonized *R. prolixus* to afford key information for enhanced understanding of vectorial capacity and human risk of exposure.

## 2. Materials and Methods

### 2.1. Insects

All insects in this study were reared in an arthropod containment level 2 (ACL2) triatomine colony with a 12 h photoperiod cycle. Microhabitat humidity experienced by the triatomines was >50% because they were housed in Nalgene primary containers (Avantor, Radnor, PA, USA) that were placed in a plastic secondary container filled with water [43]. The secondary containers sit in tubs coated with fluon (BioQuip Products, Rancho Dominguez, CA, USA) to reduce risk of escape. *Rhodnius prolixus* were obtained from Centers of Disease Control and Prevention from a colony developed from insects collected in Colombia (NR-44077, BEI Resources, Manassas, VA, USA) and were many generations removed from the wild population. *Triatoma gerstaeckeri* and *T. sanguisuga* were offspring of individuals collected from wild populations in Texas between 2017 and 2019. The laboratory generations F1 to F2 were used in the trials. All nymphs came from known *T. cruzi*-negative colonies, which were separated from adults and subsampled to confirm infection status following testing protocols described below. Triatomines were maintained on defibrinated rabbit (*Oryctolagus cuniculus domesticus*) blood (Hemostat Laboratories, Dixon, CA, USA) weekly through artificial membrane feeders (Hemotek Ltd., Blackburn, UK).

### 2.2. Guinea Pigs

Fourteen adult female guinea pigs were used in the trials. They were uniquely marked with fur pigment markers (Stoelting, Wood Dale, IL, USA) and group housed in an animal BSL-1 containment. Although *T. cruzi* infection was not an expected outcome in animals in this study, each of the guinea pigs had their blood drawn at three different time points: pre-study, mid-study, and post-study to confirm negative infection status and to facilitate their adoption at the end of the study. Animal use was approved by the Texas A&M University’s Institutional Animal Use and Care Committee (2018-0484).

We extracted DNA from 50 to 100 µL of whole blood (E.Z.N.A.^®^ Blood DNA Kit, Omega Bio-tek, Norcross, GA, USA) following the kit’s instructions. A negative control, which was 250 µL of phosphate-buffered saline (PBS) solution (VWR, Radnor, PA, USA), was included in every DNA extraction. We used real-time qPCR to detect a 166 bp fragment of the *T. cruzi* nuclear satellite DNA with the primers Cruzi 1, 2, and 3 [44,45]. In the qPCR run, water negative controls and a *T. cruzi* positive control (Sylvio-X10 CL4; ATCC 50800, American Type Culture Collection, Manassas, VA, USA) were included. Cycle threshold values of 35 or higher were considered negative for the detection of parasite DNA.

### 2.3. Parasite Culture

We obtained *T. cruzi* metacyclic trypomastigotes by gently compressing the abdomen of a naturally infected, wild-caught *T. gerstaeckeri* nymph from Frio County, TX, that had previously tested positive for *T. cruzi* discrete typing unit (DTU) TcI. We obtained *T. cruzi* epimastigotes of *T. cruzi* DTU TcIV by hemoculture of a naturally infected non-human primate from a central Texas biomedical research facility [46]. Trypanosomes were cultured in liver-infusion tryptose (LIT) media supplemented with fetal bovine serum, penicillin–streptomycin, and nystatin (Sigma-Aldrich, Darmstadt, Germany) [47,48,49]. Culture flasks were placed into an incubator at 27 °C and microscopically examined for the presence of motile trypanosomes two weeks later. Cultures were maintained by passaging in LIT media and were mixtures of abundant epimastigotes and rare trypomastigotes as determined by microscopy. The *T. cruzi* DTU in each culture was confirmed by a multiplex qPCR targeting the spliced leader intergenic region (SL-IR) from a published protocol [50]. Positive controls were from Sylvio-X10 CL4 for TcI and from a *T. cruzi*-positive triatomine from the field for TcIV. Water negative controls were also included in the qPCR runs. A CT value of <35 was considered positive for that DTU.

### 2.4. Experimental Infections and Confirmation of Infection Status

**Figure 1:**
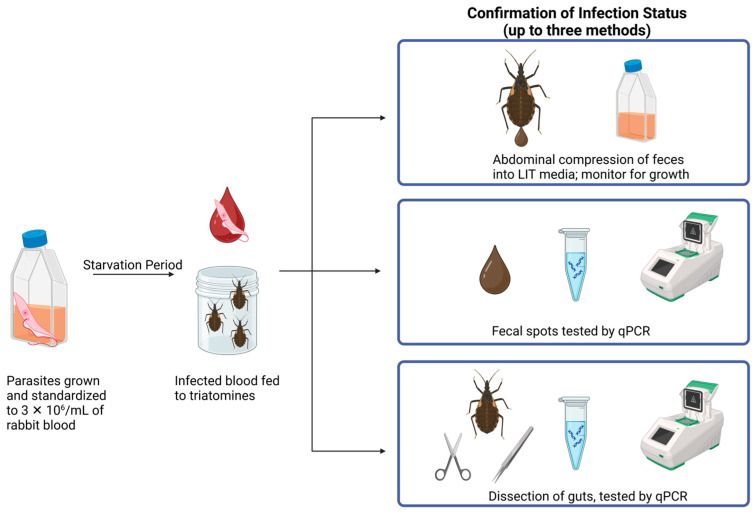
Flowchart of the methods used in experimental infections and the three methods for subsequent confirmation of infection status: abdominal compression, fecal spot testing, gut dissection testing. Illustration created in BioRender.

Experimental infections and subsequent confirmation of infection status followed the methods illustrated in Figure 1. To calculate parasite concentration of each culture, we agitated the culture flask and pipetted 10 µL of media into 90 µL of formalin (VWR, Radnor, PA, USA) and counted parasites in a hemocytometer (Reichert, Buffalo, NY, USA) to determine an approximate density while accounting for the formalin dilution. To concentrate the parasites, we centrifuged samples in microcentrifuge tubes for 10 min at 956× *g*, poured off the culture medium, and resuspended with sterile PBS solution (VWR, Radnor, PA, USA) after which the process was repeated, and the pellet was retrieved. We transferred the washed parasite pellet into a measured quantity of defibrinated rabbit blood to a final estimated concentration of 3 × 10^6^ parasites per mL of blood, a concentration similar to peak parasitemia in laboratory mice, which has a very high probability of insect infection [51,52,53,54].

Infected blood was offered to fourth and fifth instar triatomine nymphs through the Hemotek membrane feeder for two hours; ambient temperatures in the room ranged from 24 to 27 °C. These instars were chosen because of robust availability in the insect colony, ease of handling, and high visibility under the surveillance cameras used in the trials. The control groups were offered blood without parasites. The insects were group housed during the experimental feedings, where the nymphs were allowed to feed to repletion. Insects that did not feed on their pre-trial bloodmeal, or fed then molted into adults prior to use, were removed from the study. We recorded the starvation period as the number of days since the last time a bloodmeal was offered prior to use in the trials; the range was 2–6 weeks.

We used up to three different methods to confirm the infection status of insects (Figure 1). Some insects were gently compressed abdominally at 2–4 weeks post feeding on infected blood so that fecal material could be directly expelled into 5 mL of LIT culture media. The cultures were incubated at 27 °C and were checked weekly for presence of *T. cruzi.* We allowed for *T. cruzi* to grow in the cultures for one month [55] to confirm infection. Some individuals would not defecate following compression, so as an alternative we used fecal spot testing. Feces from insects voided naturally onto filter paper was cut out using sterile scissors and held individually in 50 mL conical tubes (Whatman Filter Paper, Sigma-Aldrich, Darmstadt, Germany).

DNA was extracted using the KingFisher Cell and Tissue kit (Thermo Fisher Scientific, Waltham, MA, USA), following the kit’s instructions but with a 1–2 h lysis period. Real-time qPCR was performed to detect the 166 bp fragment of *T. cruzi* DNA as described in the ‘Guinea Pigs’ section [33]. If infection status was not confirmed with one of these methods, then after their use in the trials, insects were dissected to obtain gut material, which was subjected to DNA extraction but with an overnight lysis and tested using qPCR [33]. If insects in the infected treatment groups were still alive after their use in the feeding trials, they were also abdominally compressed to further confirm their final infection status.

### 2.5. Feeding and Defecation Behavioral Trials

The trials were conducted from August 2019 to September 2020 in an ABSL2 and ACL2 negative air pressure biocontainment unit (bioBUBBLE, Fort Collins, CO, USA). Temperatures ranged from 18 to 26 °C; the mean temperature was 23.4 °C (±2.4). The hours of the day when trials were conducted ranged from 8:30 a.m. to 7:00 p.m. Experimental arenas were a 17.6 in × 11.5 in × 7.8 in, clear, polycarbonate Sous Vide container (Lipavi, UK) with the bottom surfaces lined with white bench paper and taped using white laboratory tape. One camera (YI Technology, Pudong District, Shanghai, China) was docked above each arena to allow recording of the trials. After the first several trials were performed in ambient light, two 25-watt red light bulbs were set up above the containers to allow for observations to be made with the low-light surveillance cameras. One trial is defined as a single triatomine feeding on a guinea pig, and four trials were run simultaneously. Each guinea pig was restrained into a 2 inch mesh, cotton stockinette (Rolyan, Warrenville, IL, USA) with both ends tied and secured with white duct tape to the sides of the containers. Each insect was weighed before and after the trial period and haphazardly assigned to be placed with a guinea pig for the first 60 min of the trial period.

Trials lasted for 120 min, with the first 60 min consisting of one guinea pig and one triatomine together in the arena, after which the guinea pig was removed, and the insect was observed for an additional 60 min. Insect behavioral scoring included (i) whether the insect was sedentary or walking; (ii) number of feeding attempts, feeding events, and interrupted feedings; and (iii) number of defecation events, with the color of each defecation noted. A feeding event started when an insect inserted its proboscis into the guinea pig for at least one continuous second and ended once the insect removed its proboscis and walked away from the location of the bite [56]. A probing attempt was defined when an insect repeatedly probed the guinea pig for less than one second each time. A defecation event occurred when an insect excreted either urinary or fecal material at any time in the two-hour trial period. The time at which the insect defecated was noted. Guinea pig behavioral scoring consisted of the (i) reaction to probing by an insect; (ii) movement inside the stockinette; (iii) and other general observations. In cases that an insect was still feeding at the 60 min mark, the guinea pig stayed in the arena until the insect finished feeding. Upon completion of the trial, the insects were weighed again and a unique colored marking with nail polish was painted over their lower abdomen. Any fecal spots on the bench paper from the trials were collected.

### 2.6. Statistical Analyses

We adopted the defecation index (DI) from Zeledon to allow for a standardized index of infection capacity to compare with other studies [4,6,7], where DI = (% of insects that defecated up to 10 min post feeding × average number of defecations up to 10 min post feeding)/100. We also calculated the PFDI as the time intervals between the start of an insect’s most recent bloodmeal in the arena to the time of its first defecation [22]; in the case of insects that fed and defecated multiple times, multiple PFDIs were calculated per trial. Given that some studies emphasize the importance of defecation at an interval less than 10 min [4,22,57], we also calculated percent of individuals defecating within 1 min post feeding because it is suggested that triatomines would already have moved away from its hosts after 1 min post feeding [8,57].

The volume of blood ingested was calculated using a proportion of 1 mg of weight gained after feeding equal to 1 µL of blood [56]. The percent weight gain was also calculated by dividing the ingested blood volume by the pre-trial weight of the insect [12].

We tested for differences among treatment groups using generalized estimating equation models, GEE [58]. Models were fit using the “geepack” package in R version 4.1.1. We employed GEE models given the nature of the data, where experiments had randomization constraints related to using different guinea pigs and were performed over different days, something that constrained the use of simpler regression tools that assume full replication [59]. To analyze whether triatomines fed or defecated, we employed logistic GEE models [60]. To analyze variables associated with the number of times a triatomine fed, or defecated/urinated, we employed models with a Poisson distribution [60]. To analyze the total feeding time and the post-feeding defecation intervals (PFDIs), we used a model with a Gaussian distribution [58].

In all models we considered the triatomine species and the infection status as the main explanatory variables. As covariates, we considered the illumination conditions for the experiment (with lights on or off), the estimated number of days the triatomine was starved before the experiment, and the nymphal instar. This basic structure was used in the model looking at factors associated with vector feeding; in all other models, we included additional covariates. For the model studying whether triatomines defecated or not, we considered whether triatomines fed when offered the guinea pig. For models studying the number of feedings, number of defecations, total feeding time, and PFDIs, we compared their goodness of fit considering either the initial insect weight or the pre- and post-feeding weight difference at the end of the experiment. For the model of number of defecations, we also added the number of feedings as a covariate.

For the inference, we used a sandwich estimator to obtain robust standard errors, since naïve standard errors are appropriate only when the correlation structure is correct [60]. When fitting the GEE models, we fitted alternative models that were either considered independent or correlated, also known as an exchangeable error structure, as a function of the clustering factor (guinea pig or date of trial) [58]. In the models, we considered the day of measurement or guinea pig identity as clustering factor. Among these alternatives, we chose the best model for each of the responses based on the minimization of the quasi-likelihood information criterion (QIC), a goodness of fit function that trades-off deviance and number of parameters in GEE models, and whose minimization can be used to choose the best model [61].

For each guinea pig, we recorded the number of trials the guinea pig was successfully fed on by an insect and used a GEE model to see if the size of bloodmeals would decrease over time as the guinea pigs’ immune system was primed to the insects’ salivary proteins [62,63]. Results and discussion are in the Supplementary Materials (Figure S1; Table S16).

## 3. Results

Of the 91 triatomines that were experimentally fed on *T. cruzi*-infected blood, *T. cruzi* was found via culture or PCR methods in 85 (93.4%) of them. Of the 64 triatomines that were in the control category, 63 (98.4%) were confirmed as uninfected. The single control insect that tested positive was excluded from analysis. Insects that had test results congruent with their assigned treatment groups were used for statistical analysis, which resulted in a total of 148 (95.5%) individual insects.

Out of 148 trials, 59 (40.0%) involved a triatomine that fed on guinea pigs during the trial periods (Table 1). Of those 59 insects, 42 (71.2%) defecated at least once after feeding during the observation period. Forty-one (64.1%) *T. gerstaeckeri* fed on the guinea pigs, while only eight (29.6%) *T. sanguisuga* and ten (17.5%) *R. prolixus* fed on the guinea pigs. For defecation, 33 (51.6%) *T. gerstaeckeri* defecated during the observation period regardless of feeding success, while there were 8 (29.6%) for *T. sanguisuga* and 15 (26.3%) for *R. prolixus*.

For the logistic GEE model to explore predictors of whether an insect fed or not, the best fit model had an independent correlation structure considering the guinea pig as the clustering factor (Table S1). We found the odds of *T. gerstaeckeri* feeding on a guinea pig was 9.21 times higher (*p* < 0.001) than in *R. prolixus*. In contrast, *T. sanguisuga* feeding odds were not different from those of *R. prolixus* (*p* = 0.825). If a triatomine was infected with TcI, it had one-third the odds of feeding on the guinea pig (*p* = 0.025) when compared with an uninfected triatomine (Table S2). There were no differences in feeding odds if a triatomine was infected with TcIV when compared with uninfected triatomines.

For the model to explore variables associated with whether an insect defecated, the best fit model had an independent correlation structure considering the guinea pig as the clustering factor (Table S3). We found there were no significant differences between species nor infection status, but if an insect was observed in the dark (with the red lights), it had an odds of defecating two times higher than the odds of an insect in ambient light (*p* = 0.045; Table S4). If an insect fed on the guinea pig, the odds of defecating was 17.99 times higher than that of an insect that did not feed on the guinea pig (*p* < 0.001).

### 3.1. Feeding Results

**Figure 2:**
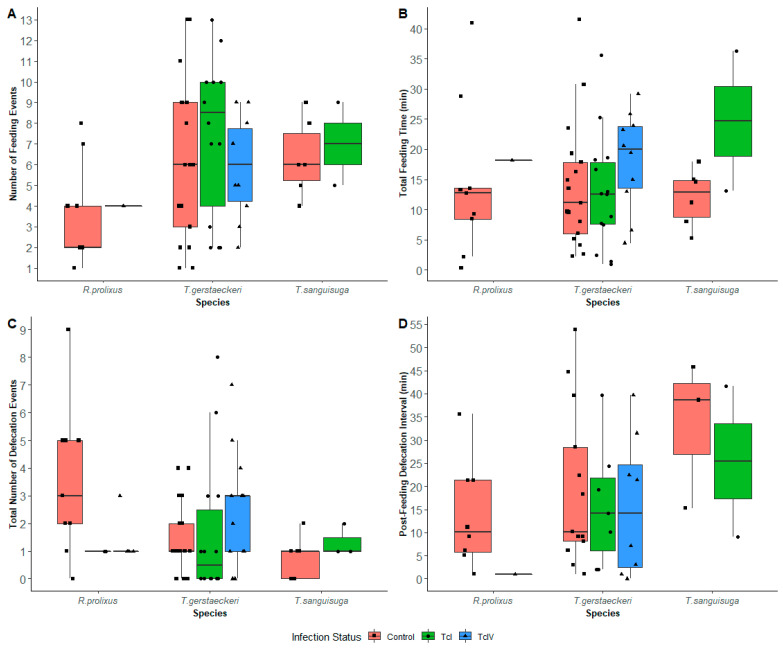
Boxplots of (**A**) number of feeding events per triatomine species: *Rhodnius prolixus* (Control, TcIV); *Triatoma gerstaeckeri* (Control, TcI, TcIV); *Triatoma sanguisuga* (Control, TcI); (**B**) total feeding times (min) per triatomine species: *R. prolixus* (Control, TcIV); *T. gerstaeckeri* (Control, TcI, TcIV); *T. sanguisuga* (Control, TcI); (**C**) total number of defecation events per triatome species: *R. prolixus* (Control, TcI, TcIV); *T. gerstaeckeri* (Control, TcI, TcIV); *T. sanguisuga* (Control, TcI); (**D**) post-feeding defecation interval (min) of the first defecation per triatomine species: *R. prolixus* (Control, TcIV); *T. gerstaeckeri* (Control, TcI, TcIV); *T. sanguisuga* (Control, TcI).

For the model exploring the number of feedings, the best fit model had an exchangeable correlation structure using the date of trial observation as the clustering factor (Table S5). Of the insects that fed, the mean number (±S.E.) of feeding events per insect within the first 60 min with the guinea pig was 6.49 (±0.56) for *T. gerstaeckeri*, 6.50 (±0.68) for *T. sanguisuga*, and 3.50 (±0.76) for *R. prolixus*. *Triatoma gerstaeckeri* had significantly more feeding events than *R. prolixus* (*p* = 0.017; Figure 2A), while there were no differences between *T. sanguisuga* and *R. prolixus* (*p =* 0.154). Aggregating species, the control group had an average of 5.34 (±0.61) feeding events per insect, while TcI had 7.44 (±0.91) and TcIV had 5.73 (±0.73) feeding events per insect. Weight gain was positively correlated (*p* < 0.001; Table S6). Out of the 59 trials where a triatomine successfully fed on a guinea pig, only one trial did not result in host agitation of the guinea pig when the triatomine fed on it. Additionally, of the 148 trials, 78 (52.7%) of the insects were noted to have probed the guinea pigs. Of these 78 insects, 58 (74.4%) also had a successful feeding event.

The best fit Gaussian model exploring total feeding time per insect had an independent correlation structure using the date of trial observation as the clustering factor (Table S7). The mean total feeding times per insect was 14.6 (±1.51) minutes for *T. gerstaeckeri,* 15.2 (±3.34) minutes for *T. sanguisuga*, and 14.8 (±3.86) minutes for *R. prolixus*. On average, *T. sanguisuga* fed 10.55 min longer than *R. prolixus* (*p* < 0.001), while total feeding times were not significantly different for *T. gerstaeckeri* (Table S8; Figure 2B). The model also showed the covariates of life stage and environment were significant: those at the fifth instar took an average of 10.43 min more to feed than those at the fourth instar (*p* < 0.001), and insects that fed in ambient light fed 4.37 min less than those with red lights (*p* = 0.021; Table S8). An insect fed for 0.065 min more for every 1 mg increase in its weight (*p* < 0.001). Aggregating species, uninfected insects fed 13.67 (±1.80) minutes per insect, TcI fed for 14.42 (±2.67) minutes, and TcIV fed for 18.13 (±2.35) minutes.

### 3.2. Defecation Results

A total of 56 (37.8%) insects defecated during the trials, and 42 of those defecated after feeding while 14 defecated without feeding during the trial. The best fit model for number of defecation events had an independent correlation structure using guinea pigs as the clustering factor (Table S9). Both *T. gerstaeckeri* and *T. sanguisuga* had, on average, fewer defecation events than *R. prolixus* (*p* < 0.001 for both) (Table S10; Figure 2C). For every 1 mg increase in an insect’s weight, there was a 6.65% increase in the number of defecations (*p* = 0.002). Infection status and other covariates did not yield significant differences in the number of defecation events. The mean (±S.E.) total number of defecation/urination events per insect were 1.80 (±0.29) for *T. gerstaeckeri*; 0.91 (±0.21) for *T. sanguisuga*; and 2.56 (±0.59) for *R. prolixus*. Regarding infection status, controls had 1.81 (±0.32) defecation events per insect; TcI had 1.53 (±0.50); and TcIV had 2.22 (±0.43).

### 3.3. Post-Feeding Defecation Intervals

We measured the post-feeding defecation interval (number of minutes from the end of a blood meal to the first defecation) using GEE models with a Gaussian distribution. The best fitted model had an independent correlation structure using guinea pigs as the clustering factor (Table S11). The average interval between first feeding to the first defecation was 9.75 (±2.52) minutes for *T. gerstaeckeri*; 20.69 (±8.98) minutes for *T. sanguisuga*; and 4.54 (±2.46) minutes for *R. prolixus*. The PFDIs for the control group was 11.82 (±3.17) minutes; TcI group was 8.19 (±3.26) minutes; and TcIV group was 6.68 (±4.02) minutes. Except for the *T. sanguisuga* (Control) group, the infection groups had PFDIs averages within 10 min (Figure 2D). Compared to *R. prolixus*, *T. gerstaeckeri*, on average, would defecate 11.45 min later post feeding (*p* < 0.001), and *T. sanguisuga* would defecate 19.52 min later post feeding (*p* < 0.001; Table S12). We did not see a significant difference between the infected and uninfected groups (TcI: *p* = 0.087; TcIV: *p* = 0.389) for the PFDIs to the first defecation.

**Figure 3:**
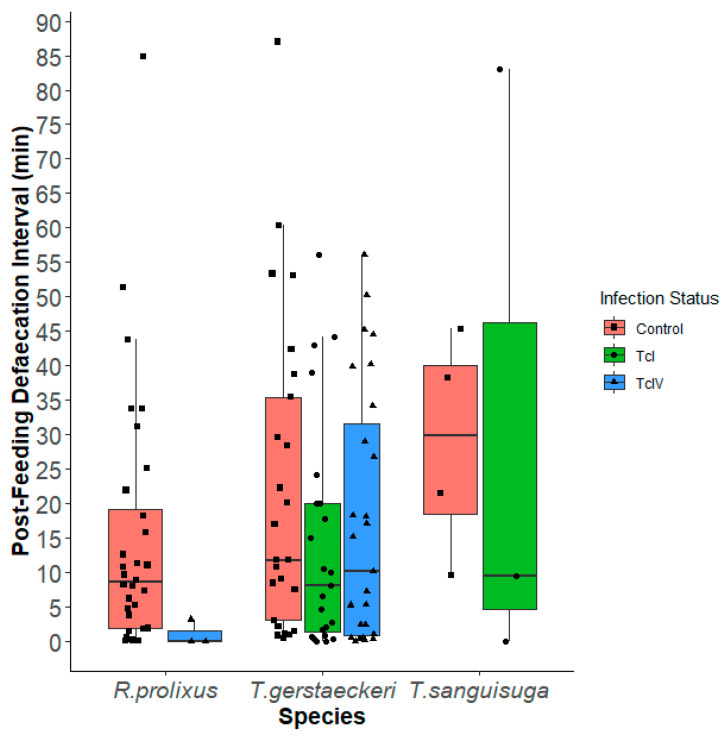
Boxplot showing each individual post-feeding defecation intervals (min) between the most recent bloodmeal and defecation per triatomine species: *Rhodnius prolixus* (Control, TcIV); *Triatoma gerstaeckeri* (Control, TcI, TcIV); *Triatoma sanguisuga* (Control, TcI). Triatomines that defecated multiple times have multiple data points in the plot.

Given many insects had multiple defecations post feeding, we fitted a model to use data on multiple PFDIs per insect with a Gaussian distribution that had an independent correlation structure using the individual insect as the clustering factor (Table S13). There was a total of 42 insects that fed and defecated which accounted for 117 individual PFDIs (Figure 3). We observed TcI-infected insects had shorter PFDIs than uninfected insects (*p* = 0.001; Table S14). In this model, *T. sanguisuga* took longer to defecate post feeding than *R. prolixus* (*p* < 0.001), and insects in ambient light took longer to defecate post feeding than those in the dark (*p* = 0.007).

**Figure 4:**
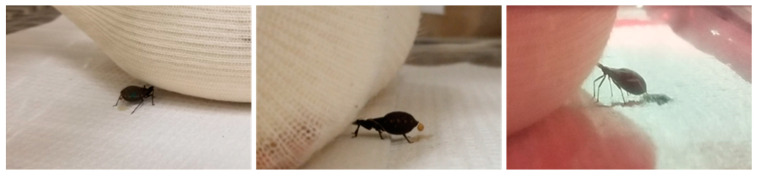
Photographs of all three species of triatomines simultaneously defecating while feeding on restrained guinea pigs. Left to right: *Triatoma gerstaeckeri, Triatoma sanguisuga, Rhodnius prolixus*.

Of the 148 triatomines that were analyzed, we observed 6 insects (4.1%) spanning all three species that simultaneously fed and defecated (Figure 4), including three that carried out so twice. A video taken during one trial showing a *T. sanguisuga* nymph simultaneously feeding and defecating is uploaded as Supplementary Material (Video S1). These insects had a total feeding time range from 13 to 40 min and defecated a total of 29 times, of which 21 defecations were within 10 min post feeding. For both *T. gerstaeckeri* and *T. sanguisuga*, only infected insects simultaneously fed and defecated, and they accounted for 14 total defecation events—10 of which were within 10 min post feeding.

### 3.4. Defecation Index

**Figure 5:**
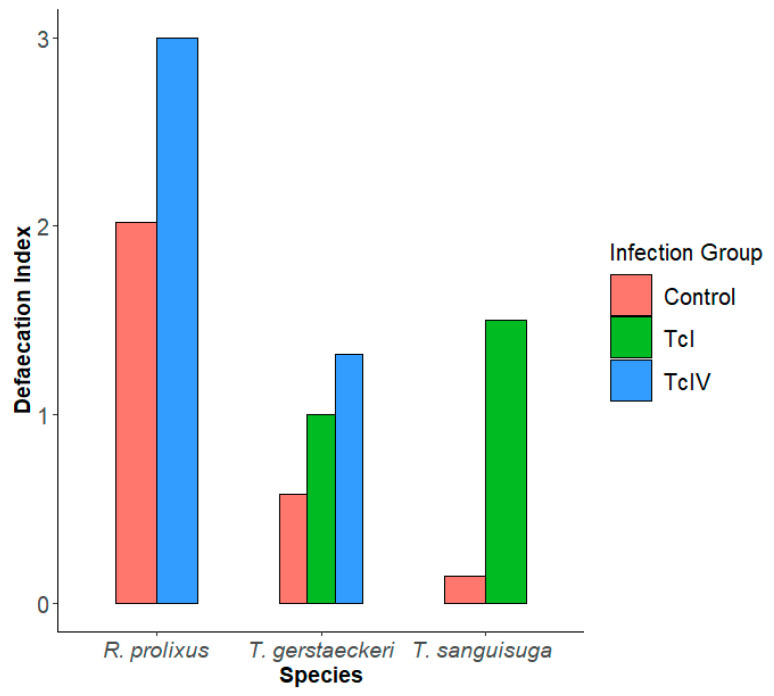
Defecation indices (DI = (% of insects that defecated up to 10 min post feeding X average number of defecations up to 10 min post feeding)/100)) of each infection group.

For *R. prolixus,* the DI for both the uninfected group (2.02) and TcIV-infected group (3.00) were higher than all treatment groups of the other species (Figure 5), but the *R. prolixus* TcIV data are from a single insect. The DI for *R. prolixus* (TcI) could not be calculated since none of the insects in that group fed on a guinea pig. The percentage of triatomines defecating within 1 min post feeding was 11 (39.3%) of *T. gerstaeckeri*, 1 (20%) of *T. sanguisuga*, and 6 (66.7%) of *R. prolixus*.

### 3.5. Weight Gain and Bloodmeal Size

We calculated the mean weight gain (mg), mean volume of blood ingested (µL), and percent weight gain for insects that fed on the guinea pigs and gained weight (Table 2). There was a total of six insects that we recorded feeding events for, but they did not gain weight and were excluded in the tabulation, so the total number of insects that fed and gained weight was *n* = 53. *T. sanguisuga* had fourth instar nymphs that fed and gained weight, while the other infection groups only had data for fifth instars. Overall, most of the infected groups gained more weight and ingested more blood. *Triatoma gerstaeckeri* (TcI) had lower mean weight gain and mean blood volume ingested but had a higher percentage weight gain than the control group. Both *T. cruzi*-infected groups (TcI and TcIV) for all three species had larger mean weight gain percentages than the control groups, apart from *R. prolixus* (TcI), since none of those insects fed.

Of those that fed and gained weight (*n* = 53), the mean bloodmeal size was 119 µL, and there was no difference in the size of insect bloodmeals that were taken on guinea pigs with different numbers of prior feeding events (*p =* 0.57; Figure S1). Our independent correlation gaussian GEE (QIC = 9.14 × 10^5^) model (Table S15), which was selected over one with a correlated structure (QIC = 9.14 × 10^5^), also showed that blood meal volume increased by 22.49 (±7.15) µL in fifth instars of all species when compared with fourth instars (*p* < 0.017; Table S16). There was an additional significant increase of 101.39 (±25.46) µL in *T. gerstaeckeri* fifth instars (*p* < 6.8 × 10^−5^) when compared with *R. prolixus*, the difference between *T. sanguisuga* and *R. prolixus* (the reference species in our analysis) not being significant (*p* > 0.66). Once the stage and species corrections were considered, the model suggested that larger triatomines ingested less blood. We found that for each mg of insect weight before blood feeding the volume of the bloodmeal decreased by −0.275 (±0.116) µL (*p* < 0.0183; Table S16).

## 4. Discussion

We observed the feeding and defecation behaviors of two epidemiologically important triatomine species found in the U.S. and compared them with a highly competent vector species from South America. Previous studies have shown *R. prolixus* to be efficient vectors for transmitting *T. cruzi* in that it defecates often and has short PFDIs [4,12,21,23], while studies of species in the U.S. have shown them to have prolonged PFDIs [6,7,9,12]. A feeding and defecation behavioral study has been performed with *T. gerstaeckeri* and *T. sanguisuga* in the U.S., showing they may be less efficient vectors than *R. prolixus* [12]; however, it has been a few decades since the study was conducted, and to our knowledge, only a few more studies observing other species that can be found in the U.S. have since been conducted [6,7,13,64]. The various behavioral metrics observed in our study for *R. prolixus* indeed support that it may be a more efficient vector than *T. gerstaeckeri* and *T. sanguisuga*. However, we found evidence for both *T. gerstaeckeri* and *T. sanguisuga* that suggest competence for stercorarian transmission of *T. cruzi* in nature.

Fewer *R. prolixus* defecated during the study period (26.3%) as compared to *T. gerstaeckeri* (51.6%); this likely results from the low rate of feeding by *R. prolixus* (17.5%) as compared to *T. gerstaeckeri* (64.1%). The lower rate of feeding may be attributed to the mixed physiological status of insects that were enrolled in the trials. In particular, we used insects in the same life stage as when they obtained their last bloodmeal; *R. prolixus* in particular may have been unmotivated to feed despite allowing for a starvation period. Whereas Buxton (1930) found a single bloodmeal per life stage was sufficient to allow molting of *R. prolixus*, Uribe (1926) found multiple feedings for each immature stage before molting. Regardless, most metrics calculated in our study result from the subset of insects that both fed and defecated from which some conclusions may be drawn.

We found that in the uninfected (control) groups, all three species had similar mean total feeding times. *Rhodnius prolixus* had fewer feeding events per insect than *T. gerstaeckeri* and *T. sanguisuga*, which may be attributed to *R. prolixus* taking a longer time to feed per feeding event. *Rhodnius prolixus* also had significantly more defecation events and shorter PFDIs than the other two species, which was expected based on previous studies [4,12,21,23] and support its role as an efficient vector of *T. cruzi*.

The influence of *T. cruzi* infection on feeding and defecation behavior is important, given most individuals are infected in nature [30,33,34]. Many studies have investigated the influence of *T. cruzi* on various aspects of triatomine behavior, such as development, fecundity, and fitness [65,66,67,68]. Some of these studies and others have seen changes in infected triatomine’s biting rates, weight gains, and defecations [11,15,22,56,69], suggesting that *T. cruzi* may modulate vector competence. One study showed infected *Mepraia spinolai* were twice more likely to bite and defecated sooner than uninfected triatomines [11], and another study showed that *T. cruzi* infection increased triatomine’s vector activity, such as host odor detection [69]. Both studies suggest manipulation by *T. cruzi* of feeding and defecation behaviors leading to increased parasite transmission. Interestingly, while we saw the *T. cruzi*-infected groups had slightly more feeding and defecation events and longer feeding times than the uninfected controls, none of these metrics were statistically different. While *T. cruzi*-infected groups tended to have shorter PFDIs to the first defecation event, this difference was not significant. However, when considering multiple, individual defecation events post feeding, TcI-infected insects had significantly shorter PFDIs than uninfected insects. This finding suggests that infection with *T. cruzi* makes a difference in the timing of multiple defecation events.

The defecation index (DI) was first proposed as a measurement to estimate an insect’s transmission capability and accounts for defecations within 10 min post feeding [4]. A higher DI generally equates to a higher capability of transmission. Zeledon calculated the DIs for fourth and fifth instars of *R. prolixus* to be 3.8 and 1.8, respectively. We saw higher DIs in both the control and TcIV groups for *R. prolixus* (2.02 and 3, respectively) compared to the North American species. The DIs we calculated for most of the *T. gerstaeckeri* and *T. sanguisuga* groups were higher than those previously reported for *Triatoma protracta* and *Triatoma rubida* [4,6,9], with one study showing adult female *T. rubida* having a DI of 1.3 [7]. Across all species, we found that the infected groups had higher DIs compared with their own control groups, providing further support that *T. cruzi* may be playing a role in increasing the vector competence of triatomines with respect to their defecation behaviors. Apart from *T. gerstaeckeri* (TcI), all infected groups had a higher mean for blood volume ingested than the control groups, similar to results found for *Triatoma rubrovaria* infected with DTU TcIV [56]. We also observed that the more blood an insect ingested—indicated by bigger weight gain—the shorter the PFDIs, corroborating that blood intake had a negative correlation with the time of appearance of the first defecation [70].

A total of six insects (4.1%) fed and defecated simultaneously, in which all three species were represented. These insects had multiple defecation events, of which a majority was within 10 min post feeding. If an insect fed to repletion, then it is possible that they will still be in close proximity to the host because the added weight may slow down its mobility [4], and the chances to defecate or urinate multiple times are high. We also observed 14 insects that defecated with no apparently successful feeding events on the guinea pig. Of these 14 insects, 6 (42.9%) were noted to have had probing attempts on the guinea pigs. We found the insects were more likely to defecate in the dark than in ambient light, which supports findings of an increased risk of parasite transmission at night or in dark nests as triatomines are likely more active to feed when their hosts are sleeping [43].

The three species we used in the trials were the same that were used in Pippin’s study in 1970 with triatomines feeding on laboratory mice and rats. His findings showed the mean total feeding times of fourth and fifth instar nymphs were 33 and 39 min, respectively, for *T. gerstaeckeri*; 25 and 31 min for *T. sanguisuga*; and 17 and 19 min for *R. prolixus* [12]. In contrast, all three species we studied had mean total feeding times of 14–15 min. Pippin also found a larger percentage of fourth and fifth instar nymphs of *R. prolixus* defecated within two minutes of feeding than that of *T. gerstaeckeri* and *T. sanguisuga*, indicating the two species were less efficient stercorarian vectors than *R. prolixus*. We did not see significant differences in determining which species was more likely to defecate, but both *T. gerstaeckeri* and *T. sanguisuga* had fewer defecation events than *R. prolixus.* Zeledon and others noted that in Pippin’s study, about a quarter of the late instar nymphs of *T. gerstaeckeri* and *T. sanguisuga* were defecated within two minutes post feeding, making them potential vectors for *T. cruzi* [71]. Applying the same method of reporting for our insects that defecated for our study, we saw 42.9% of *T. gerstaeckeri* and 20.0% of *T. sanguisuga* defecating within two minutes after feeding, which is similar to *T. sanguisuga* (23.3%) but higher than *T. gerstaeckeri* (25.0%) observed in Pippin’s study [12].

A previous study reported that *T. sanguisuga* were more likely to carry DTU TcIV [33], yet we did not have a TcIV infection group for that species. *Triatoma sanguisuga* is more challenging to colonize [72,73] than *T. gerstaeckeri* and had insufficient numbers to properly make comparisons between TcIV-infected and uninfected insects in the trials. Surprisingly, our results showed none of the *R. prolixus* (TcI) insects fed on the guinea pigs. This observation is likely due to the individuals being offered the guinea pig prior to molting, as multiple studies have shown that they only require one blood meal for each life stage [16,74], unlike some *Triatoma* species, which often take multiple bloodmeals prior to molting [75], which we observed in our *Triatoma* insects. We group housed the individuals when feeding through the artificial membrane, which is a strategy to encourage higher feeding success [76]. But then we did not track individuals through time to know exactly which ones had molted and which ones had not prior to the experiments. DTU TcI is also known to be mostly associated with *R. prolixus* [53,77,78], so due to the lack of data for that infection group, we could not assess if *T. cruzi* can influence the feeding and defecation patterns in *R. prolixus* [79].

Limitations include that there was a variation in the days since molting for the nymphs used in the experiments, and these data were not tracked so we do not know how it influences the outcome. This wide range in days since molting was selected to maximize the number of individuals available to include in the multiple groups but may have impacted the insects’ willingness to feed (Buxton 1930). Although we specifically documented the number of days since each insect was offered their last bloodmeal, which was used as the estimated starvation period, some insects may have not fed when offered blood, such that the real starvation period is longer than our estimate.

The impact of abdominal compression on subsequent triatomine behavior is not known but is not likely to have impacted our study findings. Of the 75 insects that were abdominally compressed successfully to confirm infection, less than 10% (*n* = 7) were performed before the trial; the rest were all tested only after the trial concluded. For the seven insects that were tested in this manner before their use in the trial, we allowed 2–4 weeks to elapse after they were tested and prior to their use in the trials. In all cases, the triatomines survived that time and some of these triatomines fed and defecated during their trials. Other insect behavioral studies have indeed performed abdominal compressions of insects, followed by use in behavioral trials [80].

Additionally, most of the trials were associated with documented behavioral response of the guinea pig host to the triatomine feeding (e.g., movement including leg jerking), which may have impacted the success of the feeding. However, the use of live animals in our experiments affords the benefit of being able to measure insect defecation after feeding on unaltered blood from a live host, which overcomes some of the challenges posed by the use of citrated blood [7] through a membrane feeder, the digestion of which is not expected to reflect reality. Upon testing to confirm the infection or control status of each insect used in the trials, we found a single control insect to test positive in the absence of apparent contamination (DNA extraction negative control and PCR negative control wells were clean). Data from this trial were not used in the analysis. Although this insect was taken from a colony thought to be free from infection, exposure to feces on filter paper (provided to lab-hatched nymphs to aid in development) taken from established colonies that include wild-caught insects with negative test results could have been a route of transmission, if those tests were imperfect and allowed for an infected insect to be present in the colony.

The results from this study will help to understand elements of vectorial capacity that may contribute to human Chagas disease risk. Although *T. gerstaeckeri* and *T. sanguisuga* had fewer defecation events and longer PFDIs than *R. prolixus*, a highly efficient vector, our observations in this study suggest that these two species are capable vectors of *T. cruzi* in the U.S. through the stercorarian form of biological transmission. While considering results on PDFI, DI, and the percent defecating within 1 min following feeding, *T. gerstaeckeri* appears to have higher transmission potential compared to *T. sanguisuga*. It is known that these species are mostly found in peridomestic or sylvatic settings, but there have been reports of them being encountered in homes [81] and of them being capable of domicile colonization [82]. Additionally, while adult triatomines are more likely to disperse and enter homes, nymphal instars can potentially become infected from other infected individuals (such as an infected adult) via triatomine cannibalism or coprophagy [83,84], especially in cases when a household has a high infestation of nymphal triatomines where they can aggregate. Studies investigating triatomine host utilization document human-derived bloodmeals, implying that they have contact with humans in the U.S. [85,86].

We present evidence that *T. cruzi* infection in triatomines might influence feeding and defecation behavior in ways that would facilitate *T. cruzi* transmission. We observed a decreased PDFI with *T. cruzi* infection, which was only significant in modeling data on all defecation events and not just the first defecation event as has been shown previously [4,7,11,22]. This observation warrants further research investigating the influence of *T. cruzi* on feeding and defecation behavior and mathematical models to determine the importance at the population level. Additionally, up to 11 species of triatomines exist in the U.S., and more studies should be conducted to compare the feeding and defecation behaviors among these species and from multiple geographic populations. The scientific and lay-community perspective that the defecation behavior or triatomines of the U.S. results in less efficient transmission of *T. cruzi* is only marginally supported by the observations in this current study. Instead, we hypothesize that other factors of triatomine ecology and contact with humans contributes to reduced vectorial capacity and lower disease burden in the U.S. compared to other regions in Latin America [87].

## Figures and Tables

**Table 1:** Descriptive summary of the frequency of feeding and defecation events during trials, which consisted of 60 min with guinea pigs and triatomines together followed by 60 min of insect-only observation.

Species	Infection Group	No. of Insects	No. Fed (%)	No. Defecated (%) ^a^	No. Fed + Defecated (%) ^b^	% Fed Insects That Defecated in 120 min
*T. gerstaeckeri*	Control	26	17 (65)	15 (58)	13 (50)	76
	TcI	21	14 (67)	7 (33)	7 (33)	50
	TcIV	17	10 (58)	11 (65)	8 (47)	80
*T. sanguisuga*	Control	16	6 (38)	5 (31)	3 (19)	50
	TcI	11	2 (17)	3 (25)	2 (17)	100
*R. prolixus*	Control	21	9 (47)	8 (42)	8 (42)	89
	TcI	19	0 (0)	2 (11)	0 (0)	0
	TcIV	17	1 (6)	5 (29)	1 (6)	100
Overall		148	59 (40)	56 (38)	42 (28)	71

^a^ This is the number of insects that defecated at least once in the 2 h period, including insects that defecated before feeding or did not feed at all. ^b^ This number represents insects that fed on a guinea pig either with simultaneous defecation or any defecation following feeding.

**Table 2:** Mean weight gain, mean blood volume ingested, and percent weight gain in the triatomines (*n* = 53 of 148 total triatomines) that engaged in feeding during the trial and gained weight ^a^ by *T. cruzi* infection status and life stage ^b^.

Species	Infection Group	Nymphal Instar	No. Insects That Gained Weight	Mean ± S.E. Weight Gain (mg)	Mean Blood Volume Ingested (µL)	% Weight Gain ± S.E.
*T. gerstaeckeri*	Control	5th	15	121 ± 26.5	120.7	98.8 ± 23.2
	TcI	5th	13	102 ± 27.9	102.2	104.8 ± 35.2
	TcIV	5th	8	265 ± 46.1	264.9	164.0 ± 37.0
*T. sanguisuga*	Control	4th	4	14 ± 9.0	14.2	44.9 ± 33.9
		5th	1	39	39.0	46.9
	TcI	4th	1	1.4	1.4	2.8
		5th	1	126	126.0	175
*R. prolixus*	Control	5th	9	71 ± 18.5	70.7	222.3 ± 44.4
	TcIV	5th	1	160	159.5	406.9

^a^ There were six insects that fed on guinea pigs but did not gain any weight. These data points were excluded from the calculations. ^b^ Some infection groups only had fifth instar triatomines that fed and gained weight. The *R. prolixus* (TcI) group did not have any insects that fed and gained weight.

## Data Availability

Raw data and R code are available in the Oak Trust Digital Repository: https://hdl.handle.net/1969.1/1582408. Accessed on 29 January 2025.

## Supplementary Materials

The following supporting information can be downloaded at: https://www.mdpi.com/article/10.3390/insects16020188/s1, Additional references include [88,89]. Video S1: A *Triatoma sanguisuga* nymph defecates while taking a bloodmeal from a guinea pig restrained in a cotton stockinette during a trial to measure feeding and defecation behaviors (20 s video). Table S1: Model selection for the best fit logistic generalized estimating equation model to studying whether or not a triatomine fed on a guinea pig. Table S2: Parameter estimates for the best logistic generalized estimating equations exploring whether or not a triatomine fed on a guinea pig. Table S3: Model selection for best fit logistic generalized estimating equation model to study whether or not a triatomine defecated during the trials. Table S4: Parameter estimates for the best logistic generalized estimating equation model exploring whether or not a triatomine defecated during the trials. Table S5: Model selection for the best Poisson generalized estimating equation model to study the total number of feedings on the guinea pig during the first 60 min of the trials. Table S6: Parameter estimates for the best Poisson generalized estimating equation model exploring the total number of feedings on the guinea pig during the first 60 min of the trials. Table S7: Model selection for the best Gaussian generalized estimating equation model to study the total feeding time (min) on the guinea pig. Table S8: Parameter estimates for the best Gaussian generalized estimating equation model studying the total feeding time (min) on the guinea pig. Table S9: Model selection for the best Poisson generalized estimating equation model to study the total number of defecation and urination events during the trials. Table S10: Parameter estimates of the best Poisson generalized estimating equation model studying the total number of defecation and urination events during the trials. Table S11: Model selection for the best Gaussian generalized estimating equation model to study the post-feeding defecation intervals (min) to the first defecation. Table S12: Parameter estimates of the best Gaussian generalized estimating equation model studying the post-feeding defecation intervals (min) to the first defecation. Table S13: Model selection for the best Gaussian generalized estimating equation model to study the individual post-feeding defecation intervals (min) between the most recent blood meal and defecation. Table S14: Parameter estimates of the best Gaussian generalized estimating equation model studying the individual post-feeding defecation intervals (min) between the most recent blood meal and defecation. Table S15: Model selection for the best Gaussian generalized estimating equation model to study the blood volume ingested (µL) of triatomines. Table S16: Parameter estimates of the best Gaussian generalized estimating equation model studying the blood volume ingested (µL) of triatomines. Figure S1: Bloodmeal sizes taken from guinea pigs by insects over time based on number of previous trials in which guinea pigs were fed on by triatomines. Data are only shown for the 59 insects that engaged in blood feeding during the trials. Each individual data point is a different insect that was used in the study with bars representing the mean bloodmeal size for each category. All guinea pigs were naïve to being fed on by triatomines at the beginning of the study (category zero). Each guinea pig was assigned a unique color.

## References

[1] Lent H., Wygodzinsky P. (1979). Revision of the Triatominae (Hemiptera, Reduviidae), and their significance as vectors of Chagas’ disease. Bull. AMNH.

[2] Galvão C., Guarneri A., Lorenzo M. (2021). Taxonomy. Triatominae—The Biology of Chagas Disease Vectors.

[3] Kirk M.L., Schofield C.J. (1987). Density-dependent timing of defaecation by *Rhodnius prolixus*, and its implications for the transmission of *Trypanosoma cruzi*. Trans. R. Soc. Trop. Med. Hyg..

[4] Zeledón R., Alvarado R., Jiron L.F. (1977). Observations on the feeding and defecation patterns of three triatomine species (Hemiptera: Reduviidae). Acta Trop..

[5] Canals M., Solis R., Tapia C., Ehrenfeld M., Cattan P. (1999). Comparison of some behavioral and physiological feeding parameters of *Triatoma infestans* Klug, 1834 and *Mepraia spinolai* Porter, 1934, vectors of Chagas disease in Chile. Mem. Inst. Oswaldo Cruz.

[6] Klotz S.A., Dorn P.L., Klotz J.H., Pinnas J.L., Weirauch C., Kurtz J.R., Schmidt J. (2009). Feeding behavior of triatomines from the southwestern United States: An update on potential risk for transmission of Chagas disease. Acta Trop..

[7] Reisenman C.E., Gregory T., Guerenstein P.G., Hildebrand J.G. (2011). Feeding and defecation behavior of *Triatoma rubida* (Uhler, 1894) (Hemiptera: Reduviidae) under laboratory conditions, and its potential role as a vector of Chagas disease in Arizona, USA. Am. J. Trop. Med. Hyg..

[8] Almeida C.E., Francischetti C.N., Pacheco R.S., Costa J. (2003). *Triatoma rubrovaria* (Blanchard, 1843) (Hemiptera-Reduviidae-Triatominae) III: Patterns of feeding, defecation and resistance to starvation. Mem. Inst. Oswaldo Cruz.

[9] Wood S.F. (1951). Importance of feeding and defecation times of insect vectors in transmission of Chagas’ Disease. J. Econ. Entomol..

[10] Aldana E., Lizano E., Rodríguez M., Valderrama A. (2001). Alimentación y defecación en triatominos del género *Rhodnius* (Hemiptera: Reduviidae) alimentados con sangre humana. Rev. Biol. Trop..

[11] Botto-Mahan C., Cattan P.E., Medel R. (2006). Chagas disease parasite induces behavioural changes in the kissing bug *Mepraia spinolai*. Acta Trop..

[12] Pippin W.F. (1970). The biology and vector capability of *Triatoma sanguisuga* Texana Usinger and *Triatoma gerstaeckeri* (StÅL) compared with *Rhodnius prolixus* (StÅL) (Hemiptera: Triatominae)1. J. Med. Entomol..

[13] Martinez-Ibarra J.A., Alejandre-Aguilar R., Paredes-Gonzalez E., Martinez-Silva M.A., Solorio-Cibrian M., Nogueda-Torres B., Trujillo-Contreras F., Novelo-Lopez M. (2007). Biology of three species of North American Triatominae (Hemiptera: Reduviidae: Triatominae) fed on rabbits. Mem. Inst. Oswaldo Cruz.

[14] Vieira C.B., Praça Y.R., Bentes K.L.d.S., Santiago P.B., Silva S.M.M., Silva G.D.S., Motta F.N., Bastos I.M.D., de Santana J.M., de Araújo C.N. (2018). Triatomines: Trypanosomatids, Bacteria, and Viruses Potential Vectors?. Front. Cell. Infect. Microbiol..

[15] Chacón F., Bacigalupo A., Álvarez-Duhart B., Cattan P.E., Solís R., Muñoz-San Martín C. (2022). The Parasite Load of *Trypanosoma cruzi* Modulates Feeding and Defecation Patterns of the Chagas Disease Vector *Triatoma infestans*. Microorganisms.

[16] Rabinovich J.E., Leal J.A., Feliciangeli de Piñero D. (1979). Domiciliary biting frequency and blood ingestion of the Chagas’s disease vector *Rhodnius prolixus* Ståhl (Hemiptera: Reduviidae), in Venezuela. Trans. R. Soc. Trop. Med. Hyg..

[17] Bacigalupo A., Segovia V., García A., Botto-Mahan C., Ortiz S., Solari A., Acuna-Retamar M., Torres-Pérez F., Cattan P.E. (2012). Differential Pattern of Infection of Sylvatic Nymphs and Domiciliary Adults of *Triatoma infestans* with *Trypanosoma cruzi* Genotypes in Chile. Am. Soc. Trop. Med. Hyg..

[18] Roellig D.M., Ellis A.E., Yabsley M.J. (2009). Oral transmission of *Trypanosoma cruzi* with opposing evidence for the theory of carnivory. J. Parasitol..

[19] Gürtler R.E., Cecere M.C., Lauricella M.A., Cardinal M.V., Kitron U., Cohen J.E. (2007). Domestic dogs and cats as sources of Trypanosoma cruzi infection in rural northwestern Argentina. Parasitology.

[20] Nóbrega A.A., Garcia M.H., Tatto E., Obara M.T., Costa E., Sobel J., Araujo W.N. (2009). Oral transmission of Chagas disease by consumption of açaí palm fruit, Brazil. Emerg. Infect. Dis..

[21] Dias E. (1956). Observations on defecation and contact feeding time of several South American Triatoma. Mem. Inst. Oswaldo Cruz.

[22] Pereyra N., Lobbia P.A., Mougabure-Cueto G. (2020). Effects of the infection with *Trypanosoma cruzi* on the feeding and excretion/defecation patterns of *Triatoma infestans*. Bull. Entomol. Res..

[23] Pipkin A.C. (1968). Domiciliary reduviid bugs and the epidemiology of Chagas’ disease in Panama (Hemiptera: Reduviidae: Triatominae). J. Med. Entomol..

[24] Mosquera K.D., Villacís A.G., Grijalva M.J. (2016). Life Cycle, Feeding, and Defecation Patterns of *Panstrongylus chinai* (Hemiptera: Reduviidae: Triatominae) Under Laboratory Conditions. J. Med. Entomol..

[25] Shields T.L., Walsh E.N. (1956). Kissing bug bite. AMA Arch. Derm..

[26] Vetter R. (2001). Kissing bugs (*Triatoma*) and the skin. Dermatol. Online J..

[27] Rabinovich J.E., Kitron U.D., Obed Y., Yoshioka M., Gottdenker N., Chaves L.F. (2011). Ecological patterns of blood-feeding by kissing-bugs (Hemiptera: Reduviidae: Triatominae). Mem. Inst. Oswaldo Cruz.

[28] Lynn M.K., Bossak B.H., Sandifer P.A., Watson A., Nolan M.S. (2020). Contemporary autochthonous human Chagas disease in the USA. Acta Trop..

[29] Bern C., Messenger L.A., Whitman J.D., Maguire J.H. (2019). Chagas Disease in the United States: A Public Health Approach. Clin. Microbiol. Rev..

[30] Kjos S.A., Snowden K.F., Olson J.K. (2009). Biogeography and *Trypanosoma cruzi* infection prevalence of Chagas disease vectors in Texas, USA. Vector Borne Zoonotic Dis..

[31] Bern C., Kjos S., Yabsley M.J., Montgomery S.P. (2011). *Trypanosoma cruzi* and Chagas’ disease in the United States. Clin. Microbiol. Rev..

[32] Sandoval-Ruiz C.A., Cervantesperedo L., Mendoza-Palmero F.S., Ibanez-Bernal S. (2012). The Triatominae (Hemiptera: Heteroptera: Reduviidae) of Veracruz, Mexico: Geographic distribution, taxonomic redescriptions, and a key. Zootaxa.

[33] Curtis-Robles R., Auckland L.D., Snowden K.F., Hamer G.L., Hamer S.A. (2018). Analysis of over 1500 triatomine vectors from across the US, predominantly Texas, for *Trypanosoma cruzi* infection and discrete typing units. Infect Genet. Evol..

[34] Kjos S.A., Marcet P.L., Yabsley M.J., Kitron U., Snowden K.F., Logan K.S., Barnes J.C., Dotson E.M. (2013). Identification of bloodmeal sources and *Trypanosoma cruzi* infection in triatomine bugs (Hemiptera: Reduviidae) from residential settings in Texas, the United States. J. Med. Entomol..

[35] Waleckx E., Suarez J., Richards B., Dorn P.L. (2014). *Triatoma sanguisuga* blood meals and potential for Chagas disease, Louisiana, USA. Emerg. Infect. Dis..

[36] Garcia M.N., Burroughs H., Gorchakov R., Gunter S.M., Dumonteil E., Murray K.O., Herrera C.P. (2017). Molecular identification and genotyping of Trypanosoma cruzi DNA in autochthonous Chagas disease patients from Texas, USA. Infect. Genet. Evol..

[37] Flores-López C.A., Mitchell E.A., Reisenman C.E., Sarkar S., Williamson P.C., Machado C.A. (2022). Phylogenetic diversity of two common Trypanosoma cruzi lineages in the Southwestern United States. Infect. Genet. Evol..

[38] Zeledón R., Rabinovich J.E. (1981). Chagas Disease: An Ecological Appraisal With Special Emphasis on its Insect Vectors. Annu. Rev. Entomol..

[39] Rassi A., Rassi A., Marin-Neto J.A. (2010). Chagas disease. Lancet.

[40] Waleckx E., Gourbière S., Dumonteil E. (2015). Intrusive versus domiciliated triatomines and the challenge of adapting vector control practices against Chagas disease. Mem. Inst. Oswaldo Cruz.

[41] Buxton P.A. (1930). The biology of a blood-sucking bug, *Rhodnius prolixus*. Trans. R. Entomol. Soc. Lond..

[42] Uribe C. (1926). On the biology and life history of *Rhodnius prolixus* Stahl. J. Parasitol..

[43] Wormington J.D., Gillum C., Meyers A.C., Hamer G.L., Hamer S.A. (2018). Daily activity patterns of movement and refuge use in *Triatoma gerstaeckeri* and *Rhodnius prolixus* (Hemiptera: Reduviidae), vectors of the Chagas disease parasite. Acta Trop..

[44] Hodo C.L., Bertolini N.R., Bernal J.C., VandeBerg J.L., Hamer S.A. (2017). Lack of *Trypanosoma cruzi* infection in urban roof rats (*Rattus rattus*) at a Texas facility housing naturally infected nonhuman primates. J. Am. Assoc. Lab. Anim. Sci..

[45] Piron M., Fisa R., Casamitjana N., López-Chejade P., Puig L., Vergés M., Gascón J., Prat J.G.i., Portús M., Sauleda S. (2007). Development of a real-time PCR assay for *Trypanosoma cruzi* detection in blood samples. Acta Trop..

[46] Hodo C.L., Wilkerson G.K., Birkner E.C., Gray S.B., Hamer S.A. (2018). *Trypanosoma cruzi* transmission among captive nonhuman primates, wildlife, and vectors. Ecohealth.

[47] Fernandes J.F., Castellani O. (1966). Growth characteristics and chemical composition of *Trypanosoma cruzi*. Exp. Parasitol..

[48] Sadigursky M., Brodskyn C.I. (1986). A new liquid medium without blood and serum for culture of hemoflagellates. Am. J. Trop. Med. Hyg..

[49] Camargo E.P. (1964). Growth and differentiation in *Trypanosoma cruzi.* I. Origin of metacyclic trypanosomes in liquid media. Rev. Inst. Med. Trop. Sao Paulo.

[50] Cura C.I., Duffy T., Lucero R.H., Bisio M., Péneau J., Jimenez-Coello M., Calabuig E., Gimenez M.J., Valencia Ayala E., Kjos S.A. (2015). Multiplex real-time PCR assay using TaqMan probes for the identification of *Trypanosoma cruzi* DTUs in biological and clinical samples. PLOS Neglected Trop. Dis..

[51] Bice D.E., Zeledon R. (1970). Comparison of infectivity of strains of *Trypanosoma cruzi* (Chagas, 1909). J. Parasitol..

[52] Mejia-Jaramillo A.M., Pena V.H., Triana-Chavez O. (2009). *Trypanosoma cruzi*: Biological characterization of lineages I and II supports the predominance of lineage I in Colombia. Exp. Parasitol..

[53] Peterson J.K., Graham A.L., Dobson A.P., Chavez O.T. (2015). *Rhodnius prolixus* life history outcomes differ when infected with different *Trypanosoma cruzi* I strains. Am. J. Trop. Med. Hyg..

[54] Añez N., Martens M.L., Romero M., Crisante G. (2011). *Trypanosoma cruzi* primary infection prevents severe re-infection in mice. Boletín Malariol. Y Salud Ambient..

[55] Fellet M.R., Lorenzo M.G., Elliot S.L., Carrasco D., Guarneri A.A. (2014). Effects of infection by *Trypanosoma cruzi* and *Trypanosoma rangeli* on the reproductive performance of the vector *Rhodnius prolixus*. PLoS ONE.

[56] Verly T., Costa S., Lima N., Mallet J., Odencio F., Pereira M., Moreira C.J.C., Britto C., Pavan M.G. (2020). Vector competence and feeding-excretion behavior of *Triatoma rubrovaria* (Blanchard, 1843) (Hemiptera: Reduviidae) infected with *Trypanosoma cruzi* TcVI. PLoS Negl. Trop. Dis..

[57] Nogueda-Torres B., Martinez-Ibarra J.A., Barboza-Lopez M., Montanez-Valdez O.D., Michel-Parra J.G. (2022). Biological Parameters of Two *Triatoma protracta* Subspecies (Hemiptera: Reduviidae). J. Med. Entomol..

[58] Venables W.N., Ripley B.D. (2002). Modern Applied Statistics with S.

[59] Chaves L.F. (2010). An Entomologist Guide to Demystify Pseudoreplication: Data Analysis of Field Studies With Design Constraints. J. Med. Entomol..

[60] Faraway J.J. (2006). Extending the Linear Model with R: Generalized Linear, Mixed Effects and Nonparametric Regression Models.

[61] Pan W. (2001). Akaike’s Information Criterion in Generalized Estimating Equations. Biometrics.

[62] Schwarz A., Sternberg J.M., Johnston V., Medrano-Mercado N., Anderson J.M., Hume J.C., Valenzuela J.G., Schaub G.A., Billingsley P.F. (2009). Antibody responses of domestic animals to salivary antigens of *Triatoma infestans* as biomarkers for low-level infestation of triatomines. Int. J. Parasitol..

[63] Dornakova V., Salazar-Sanchez R., Borrini-Mayori K., Carrion-Navarro O., Levy M.Z., Schaub G.A., Schwarz A. (2014). Characterization of guinea pig antibody responses to salivary proteins of *Triatoma infestans* for the development of a triatomine exposure marker. PLoS Negl. Trop. Dis..

[64] Martinez-Ibarra J.A., Paredes-Gonzalez E., Licon-Trillo A., Montanez-Valdez O.D., Rocha-Chavez G., Nogueda-Torres B. (2012). The biology of three Mexican-American species of Triatominae (Hemiptera: Reduviidae): *Triatoma recurva*, *Triatoma protracta* and *Triatoma rubida*. Mem. Inst. Oswaldo Cruz.

[65] Cordero-Montoya G., Flores-Villegas A.L., Salazar-Schettino P.M., Vences-Blanco M.O., Rocha-Ortega M., Gutierrez-Cabrera A.E., Rojas-Ortega E., Cordoba-Aguilar A. (2019). The cost of being a killer’s accomplice: *Trypanosoma cruzi* impairs the fitness of kissing bugs. Parasitol. Res..

[66] Elliot S.L., Rodrigues Jde O., Lorenzo M.G., Martins-Filho O.A., Guarneri A.A. (2015). *Trypanosoma cruzi*, etiological agent of Chagas disease, is virulent to its triatomine vector *Rhodnius prolixus* in a temperature-dependent manner. PLoS Negl. Trop. Dis..

[67] Villalobos G., Nava-Bolanos A., De Fuentes-Vicente J.A., Tellez-Rendon J.L., Huerta H., Martinez-Hernandez F., Rocha-Ortega M., Gutierrez-Cabrera A.E., Ibarra-Cerdena C.N., Cordoba-Aguilar A. (2019). A reduction in ecological niche for *Trypanosoma cruzi*-infected triatomine bugs. Parasit. Vectors.

[68] Schaub G.A. (1988). Developmental time and mortality of larvae of *Triatoma infestans* infected with *Trypanosoma cruzi*. Trans. R. Soc. Trop. Med. Hyg..

[69] Ramirez-Gonzalez M.G., Flores-Villegas A.L., Salazar-Schettino P.M., Gutierrez-Cabrera A.E., Rojas-Ortega E., Cordoba-Aguilar A. (2019). Zombie bugs? Manipulation of kissing bug behavior by the parasite *Trypanosoma cruzi*. Acta Trop..

[70] Trumper E.V., Gorla D.E. (1991). Density-dependent timing of defaecation by *Triatoma infestans*. Trans. R. Soc. Trop. Med. Hyg..

[71] Zeledón, Beard C., Dias J.C., Leiby D.A., Dorn P., Coura J.R. (2012). An Appraisal of the Status of Chagas Disease in the United States.

[72] Hays K.L. (1966). Some habitat requirements for *Triatoma sanguisuga* (Le Conte) (Hemiptera; Reduviidae). J. Alabama Acad. Sci..

[73] Hays K.L. (1965). Longevity, Fecundity, and Food Intake of Adult *Triatoma sanguisuga* (Leconte) (Hemiptera: Triatominae)1. J. Med. Entomol..

[74] Wigglesworth V.B. (1934). Memoirs: The Physiology of Ecdysis in *Rhodnius prolixus* (Hemiptera). II. Factors controlling Moulting and ‘Metamorphosis’. J. Cell Sci..

[75] Soares R.P., das Graças Evangelista L., Laranja L.S., Diotaiuti L. (2000). Population dynamics and feeding behavior of *Triatoma brasiliensis* and *Triatoma pseudomaculata*, main vectors of chagas disease in Northeastern Brazil. Mem. Inst. Oswaldo Cruz.

[76] de Azambuja P., Garcia E.S., Crampton J.M., Beard C.B., Louis C. (1997). Care and maintenance of triatomine colonies. The Molecular Biology of Insect Disease Vectors: A Methods Manual.

[77] Zingales B., Miles M.A., Campbell D.A., Tibayrenc M., Macedo A.M., Teixeira M.M., Schijman A.G., Llewellyn M.S., Lages-Silva E., Machado C.R. (2012). The revised *Trypanosoma cruzi* subspecific nomenclature: Rationale, epidemiological relevance and research applications. Infect. Genet. Evol..

[78] Carrasco H.J., Segovia M., Llewellyn M.S., Morocoima A., Urdaneta-Morales S., Martínez C., Martínez C.E., Garcia C., Rodríguez M., Espinosa R. (2012). Geographical distribution of *Trypanosoma cruzi* genotypes in Venezuela. PLoS Neglected Trop. Dis..

[79] Takano-Lee M., Edman J.D. (2002). Lack of manipulation of *Rhodnius prolixus* (Hemiptera: Reduviidae) vector competence by *Trypanosoma cruzi*. J. Med. Entomol..

[80] Jiménez-Santiago B., Flores-Villegas A.L., Cruz-Esteban S., Cabrera-Bravo M., Toriello C. (2024). *Trypanosoma cruzi* infection enhances olfactory response in *Triatoma pallidipennis* Stål (Hemiptera: Triatominae) to compounds potentially useful for insect control. Med. Vet. Entomol..

[81] Curtis-Robles R., Wozniak E.J., Auckland L.D., Hamer G.L., Hamer S.A. (2015). Combining public health education and disease ecology research: Using citizen science to assess Chagas disease entomological risk in Texas. PLoS Negl. Trop. Dis..

[82] Edward J.W., Gena L., Rodion G., Hasanat A., Ellen D., Blake S., Sahotra S., Kristy O.M. (2015). The Biology of the Triatomine Bugs Native to South Central Texas and Assessment of the Risk They Pose for Autochthonous Chagas Disease Exposure. J. Parasitol..

[83] Ryckman R.E. (1951). Recent observations of cannibalism in Triatoma (Hemiptera: Reduviidae). J. Parasitol..

[84] Schaub G.A. (1988). Direct transmission of *Trypanosoma cruzi* between vectors of Chagas’ disease. Acta Trop..

[85] Balasubramanian S., Curtis-Robles R., Chirra B., Auckland L.D., Mai A., Bocanegra-Garcia V., Clark P., Clark W., Cottingham M., Fleurie G. (2022). Characterization of triatomine bloodmeal sources using direct Sanger sequencing and amplicon deep sequencing methods. Sci. Rep..

[86] Gorchakov R., Trosclair L.P., Wozniak E.J., Feria P.T., Garcia M.N., Gunter S.M., Murray K.O. (2016). *Trypanosoma cruzi* Infection Prevalence and Bloodmeal Analysis in Triatomine Vectors of Chagas Disease From Rural Peridomestic Locations in Texas, 2013–2014. J. Med. Entomol..

[87] Gürtler R., Fernández M., Cardinal M.V. (2021). Eco-Epidemiology of Vector-Borne Transmission of Trypanosoma cruzi in Domestic Habitats.

[88] Sant’Anna M.R.V., Soares A.C., Araujo R.N., Gontijo N.F., Pereira M.H. (2017). Triatomines (Hemiptera, Reduviidae) blood intake: Physical constraints and biological adaptations. J. Insect Physiol..

[89] Randolph S.E. (1979). Population regulation in ticks: The role of acquired resistance in natural and unnatural hosts. Parasitology.

