# Supplementary material for: Comparative Feeding and Defecation Behaviors of Trypanosoma cruzi-Infected and Uninfected Triatomines (Hemiptera: Reduviidae) from the Americas
Source: Insects. 2025 Feb 10;16(2):188. doi: 10.3390/insects16020188 (PMC11856564; doi:10.3390/insects16020188)
Supplement: Supplementary file 1 [file insects-16-00188-s001.zip › insects-3415646-supplementary.pdf]

Supplementary Materials

Supplemental Video S1: A *Triatoma sanguisuga* nymph defecates while taking a bloodmeal from a guinea pig restrained in a cotton stockinette during a trial to measure feeding and defecation behaviors (20 second video).

Table S1: Model selection for the best fit logistic generalized estimating equation model to studying whether or not a triatomine fed on a guinea pig

Correlation Structure	Cluster	Variables	QIC
Independent	Guinea Pig	Illumination Environment, Starvation Period, Life Stage, Triatomine Species, *T.cruzi* DTU	173.52
Independent	Date of Trial	Illumination Environment, Starvation Period, Life Stage, Triatomine Species, *T.cruzi* DTU	177.15
Exchangeable	Guinea Pig	Illumination Environment, Starvation Period, Life Stage, Triatomine Species, *T.cruzi* DTU	179.78
Exchangeable	Date of Trial	Illumination Environment, Starvation Period, Life Stage, Triatomine Species, *T.cruzi* DTU	178.27

QIC: Quasi-likelihood Information Criterion

Table S2: Parameter estimates for the best logistic generalized estimating equations exploring whether or not a triatomine fed on a guinea pig

Parameter	Odds Ratio	Estimate (± S.E.)	*P*-value
Intercept—Lights Off, 4th Instar, *R. prolixus*, Uninfected	1.00	—	—
Illumination Environment—Lights On	1.68	0.52 (± 0.40)	0.196
Starvation Period	1.00	0.001 (± 0.01)	0.911
Life Stage—5th Instar	0.48	-0.73 (± 0.78)	0.350
T. gerstaeckeri	9.21	2.22 (± 0.44)	<0.001*
T. sanguisuga	1.17	0.16 (± 0.72)	0.825
TcI Infected	0.34	-1.09 (± 0.49)	0.025*
TcIV Infected	0.34	-0.94 (± 0.58)	0.109

*Statistically significant (*P*<0.05)

Table S3: Model selection for best fit logistic generalized estimating equation model to study whether or not a triatomine defecated during the trials

Correlation Structure	Cluster	Variables	QIC
Independent	Guinea Pig	Fed, Illumination Environment, Starvation Period, Life Stage, Triatomine Species, *T. cruzi* DTU	151.43
Independent	Date of Trial	Fed, Illumination Environment, Starvation Period, Life Stage, Triatomine Species, *T. cruzi* DTU	151.76
Exchangeable	Guinea Pig	Fed, Illumination Environment, Starvation Period, Life Stage, Triatomine Species, *T. cruzi* DTU	153.16
Exchangeable	Date of Trial	Fed, Illumination Environment, Starvation Period, Life Stage, Triatomine Species, *T. cruzi* DTU	153.03

QIC: Quasi-likelihood Information Criterion

Table S4: Parameter estimates for the best logistic generalized estimating equation model exploring whether or not a triatomine defecated during the trials

Parameter	Odds Ratio	Estimate (± S.E.)	*P*-value
Intercept—Lights Off, 4th Instar, *R. prolixus*, Uninfected	1.00	—	—
Fed	17.99	2.89 (± 0.61)	<0.001*
Illumination Environment—Lights On	0.52	-0.65 (± 0.32)	0.045*
Starvation Period	0.98	-0.02 (± 0.01)	0.213
Life Stage—5th Instar	2.75	1.01 (± 0.64)	0.116
T. gerstaeckeri	1.16	0.15 (± 0.44)	0.725

T. sanguisuga	1.86	0.62 (± 0.59)	0.294
TcI Infected	0.64	-0.45 (± 0.58)	0.444
TcIV Infected	1.99	0.69 (± 0.43)	0.106

*Statistically significant (*P*<0.05)

Table S5: Model selection for the best Poisson generalized estimating equation model to study the total number of feedings on the guinea pig during the first 60 minutes of the trials

Correlation Structure	Cluster	Variables	QIC
Independent	Guinea Pig	Illumination Environment, Starvation Period, Initial Weight, Life Stage, Triatomine Species, *T. cruzi* DTU	-453.02
Independent	Date of Trial	Illumination Environment, Starvation Period, Initial Weight, Life Stage, Triatomine Species, *T. cruzi* DTU	-455.39
Exchangeable	Guinea Pig	Illumination Environment, Starvation Period, Initial Weight, Life Stage, Triatomine Species, *T. cruzi* DTU	-453.22
Exchangeable	Date of Trial	Illumination Environment, Starvation Period, Initial Weight, Life Stage, Triatomine Species, *T. cruzi* DTU	-453.23
Independent	Guinea Pig	Illumination Environment, Starvation Period, Weight Change, Life Stage, Triatomine Species, *T. cruzi* DTU	-468.15
Independent	Date of Trial	Illumination Environment, Starvation Period, Weight Change, Life Stage, Triatomine Species, *T. cruzi* DTU	-469.60
Exchangeable	Guinea Pig	Illumination Environment, Starvation Period, Weight Change, Life Stage, Triatomine Species, *T. cruzi* DTU	-468.73
Exchangeable	Date of Trial	Illumination Environment, Starvation Period, Weight Change, Life Stage, Triatomine Species, *T. cruzi* DTU	-469.95

QIC: Quasi-likelihood Information Criterion

Table S6: Parameter estimates for the best Poisson generalized estimating equation model exploring the total number of feedings on the guinea pig during the first 60 minutes of the trials

Parameter	Proportional # Increase	Estimate (± S.E.)	*P*-value
Intercept—Lights Off, 4th Instar, *R. prolixus*, Uninfected	1.00	—	—
Starvation Period	1.00	0.00 (± 0.005)	0.991
Illumination Environment—Lights On	1.02	0.02 (± 0.18)	0.899
Insect's Weight Change (g)	2.78	2.76 (± 0.67)	<0.001*
Life Stage—5th Instar	0.89	-0.12 (± 0.43)	0.786
T. gerstaeckeri	2.03	0.71 (± 0.30)	0.017*
T. sanguisuga	1.81	0.60 (± 0.42)	0.154
TcI Infected	1.16	0.15 (± 0.22)	0.495
TcIV Infected	0.56	-0.57 (± 0.34)	0.093

*Statistically significant (*P*<0.05)

Table S7: Model selection for the best Gaussian generalized estimating equation model to study the total feeding time (min) on the guinea pig

Correlation Structure	Cluster	Variables	QIC
Independent	Guinea Pig	Illumination Environment, Starvation Period, Initial Weight, Life Stage, Triatomine Species, *T. cruzi* DTU	4971.97

Independent	Date of Trial	Illumination Environment, Starvation Period, Initial Weight, Life Stage, Triatomine Species, *T. cruzi* DTU	4971.95
Exchangeable	Guinea Pig	Illumination Environment, Starvation Period, Initial Weight, Life Stage, Triatomine Species, *T. cruzi* DTU	5090.40
Exchangeable	Date of Trial	Illumination Environment, Starvation Period, Initial Weight, Life Stage, Triatomine Species, *T. cruzi* DTU	5113.30
Independent	Guinea Pig	Illumination Environment, Starvation Period, Weight Change, Life Stage, Triatomine Species, *T. cruzi* DTU	2869.63
Independent	Date of Trial	Illumination Environment, Starvation Period, Weight Change, Life Stage, Triatomine Species, *T. cruzi* DTU	2868.98
Exchangeable	Guinea Pig	Illumination Environment, Starvation Period, Weight Change, Life Stage, Triatomine Species, *T. cruzi* DTU	2879.04
Exchangeable	Date of Trial	Illumination Environment, Starvation Period, Weight Change, Life Stage, Triatomine Species, *T. cruzi* DTU	2887.92

QIC: Quasi-likelihood Information Criterion

Table S8: Parameter estimates for the best Gaussian generalized estimating equation model studying the total feeding time (min) on the guinea pig

Parameter	Estimate (± S.E.)	*P*-value
Intercept—Lights Off, 4th Instar, *R. prolixus*, Uninfected	2.47 (± 3.71)	0.506
Starvation Period	-0.09 (± 0.05)	0.077
Illumination Environment—Lights On	-4.36 (± 1.89)	0.021*
Insect's Weight Change (g)	64.92 (± 11.89)	<0.001*
Life Stage—5th Instar	10.43 (± 2.70)	<0.001*
T. gerstaeckeri	-3.14 (± 3.50)	0.370
T. sanguisuga	10.55 (± 2.96)	<0.001*
TcI Infected	3.86 (± 2.65)	0.145
TcIV Infected	-3.46 (± 2.38)	0.146

*Statistically significant (P<0.05)

Table S9: Model selection for the best Poisson generalized estimating equation model to study the total number of defecation and urination events during the trials

Correlation Structure	Cluster	Variables	QIC
Independent	Guinea Pig	Illumination Environment, Starvation Period, Number of Feedings, Initial Weight, Life Stage, Triatomine Species, *T. cruzi* DTU	85.85
Independent	Date of Trial	Illumination Environment, Starvation Period, Number of Feedings, Initial Weight, Life Stage, Triatomine Species, *T. cruzi* DTU	91.60
Exchangeable	Guinea Pig	Illumination Environment, Starvation Period, Number of Feedings, Initial Weight, Life Stage, Triatomine Species, *T. cruzi* DTU	94.15
Exchangeable	Date of Trial	Illumination Environment, Starvation Period, Number of Feedings, Initial Weight, Life Stage, Triatomine Species, *T. cruzi* DTU	89.86
Independent	Guinea Pig	Illumination Environment, Starvation Period, Number of Feedings, Weight Change, Life Stage, Triatomine Species, *T. cruzi* DTU	61.00
Independent	Date of Trial	Illumination Environment, Starvation Period, Number of Feedings, Weight Change, Life Stage, Triatomine Species, *T. cruzi* DTU	64.89

Exchangeable	Guinea Pig	Illumination Environment, Starvation Period, Number of Feedings, Weight Change, Life Stage, Triatomine Species, *T. cruzi* DTU	69.08
Exchangeable	Date of Trial	Illumination Environment, Starvation Period, Number of Feedings, Weight Change, Life Stage, Triatomine Species, *T. cruzi* DTU	63.19

QIC: Quasi-likelihood Information Criterion

Table S10: Parameter estimates of the best Poisson generalized estimating equation model studying the total number of defecation and urination events during the trials

Parameter	Proportional Increase	#	Estimate (± S.E.)	*P*-value
Intercept—Lights Off, 4th Instar, *R. prolixus*, Uninfected	1.00		—	—
Number of Feedings	1.07		0.07 (± 0.04)	0.080
Starvation Period	1.00		0.00 (± 0.004)	0.950
Illumination Environment—Lights On	1.19		0.18 (± 0.13)	0.169
Insect's Weight Change (g)	67.49		4.21 (± 1.38)	0.002*
Life Stage—5th Instar	1.16		0.14 (± 0.22)	0.507
T. gerstaeckeri	0.37		-1.01 (± 0.15)	<0.001*
T. sanguisuga	0.39		-0.94 (± 0.14)	<0.001*
TcI Infected	0.85		-0.16 (± 0.26)	0.549
TcIV Infected	1.06		0.06 (± 0.20)	0.755

*Statistically significant (*P*<0.05)

Table S11: Model selection for the best Gaussian generalized estimating equation model to study the post-feeding defecation intervals (min) to the first defecation

Correlation Structure	Cluster	Variables	QIC
Independent	Guinea Pig	Illumination Environment, Starvation Period, Initial Weight, Life Stage, Triatomine Species, *T. cruzi* DTU	6422.22
Independent	Date of Trial	Illumination Environment, Starvation Period, Initial Weight, Life Stage, Triatomine Species, *T. cruzi* DTU	6426.44
Exchangeable	Guinea Pig	Illumination Environment, Starvation Period, Initial Weight, Life Stage, Triatomine Species, *T. cruzi* DTU	6587.01
Exchangeable	Date of Trial	Illumination Environment, Starvation Period, Initial Weight, Life Stage, Triatomine Species, *T. cruzi* DTU	6437.36
Independent	Guinea Pig	Illumination Environment, Starvation Period, Weight Change, Life Stage, Triatomine Species, *T. cruzi* DTU	5243.38
Independent	Date of Trial	Illumination Environment, Starvation Period, Weight Change, Life Stage, Triatomine Species, *T. cruzi* DTU	5248.51
Exchangeable	Guinea Pig	Illumination Environment, Starvation Period, Weight Change, Life Stage, Triatomine Species, *T. cruzi* DTU	5400.46
Exchangeable	Date of Trial	Illumination Environment, Starvation Period, Weight Change, Life Stage, Triatomine Species, *T. cruzi* DTU	5359.62

QIC: Quasi-likelihood Information Criterion

Table S12: Parameter estimates of the best Gaussian generalized estimating equation model studying the post-feeding defecation intervals (min) to the first defecation

Parameter	Estimate (± S.E.)	*P*-value
Intercept—Lights Off, 4th Instar, *R. prolixus*, Uninfected	0.19 (± 7.37)	0.980
Starvation Period	0.18 (± 0.13)	0.384

Illumination Environment—Lights On	3.13 (± 2.37)	0.187
Insect's Weight Change (g)	-58.98 (± 12.08)	<0.001*
Life Stage—5th Instar	6.10 (± 7.56)	0.420
T. gerstaeckeri	11.45 (± 2.08)	<0.001*
T. sanguisuga	19.52 (± 1.60)	<0.001*
TcI Infected	-8.26 (± 4.83)	0.087
TcIV Infected	2.87 (± 3.33)	0.389

*Statistically significant (*P*<0.05)

Table S13: Model selection for the best Gaussian generalized estimating equation model to study the individual post-feeding defecation intervals (min) between the most recent blood meal and defecation

Correlation Structure	Cluster	Variables	QIC
Independent	Unique Insect Code	Illumination Environment, Starvation Period, Initial Weight, Life Stage, Triatomine Species, *T. cruzi* DTU,	38269.28
Exchangeable	Unique Insect Code	Illumination Environment, Starvation Period, Initial Weight, Life Stage, Triatomine Species, *T. cruzi* DTU,	38271.61
Independent	Unique Insect Code	Illumination Environment, Starvation Period, Weight Change, Life Stage, Triatomine Species, *T. cruzi* DTU,	38992.45
Exchangeable	Unique Insect Code	Illumination Environment, Starvation Period, Weight Change, Life Stage, Triatomine Species, *T. cruzi* DTU,	39086.63

QIC: Quasi-likelihood Information Criterion

Table S14: Parameter estimates of the best Gaussian generalized estimating equation model studying the individual post-feeding defecation intervals (min) between the most recent blood meal and defecation

Parameter	Estimate (± S.E.)	*P*-value
Intercept—Lights Off, 4th Instar, *R. prolixus*, Uninfected	-9.98 (± 9.41)	0.289
Starvation Period	0.21 (± 0.17)	0.223
Illumination Environment—Lights On	13.45 (± 4.97)	0.007*
Initial Weight (g)	56.7 (± 30.94)	0.067
Life Stage—5th Instar	15.06 (± 9.47)	0.112
T. gerstaeckeri	1.85 (± 4.92)	0.707
T. sanguisuga	26.50 (± 7.60)	<0.001*
TcI Infected	-18.72 (± 5.80)	0.001*
TcIV Infected	-5.42 (± 4.26)	0.204

*Statistically significant (*P*<0.05)

Table S15: Model selection for the best Gaussian generalized estimating equation model to study the blood volume ingested (μL) of triatomines

Correlation Structure	Cluster	Variables	QIC
Independent[a]	Guinea Pig	Triatomine Species, Life Stage, Number of Trials Guinea Pig was Previously Fed On, Initial Weight	9.14e+05
Exchangeable[a]	Guinea Pig	Triatomine Species, Life Stage, Number of Trials Guinea Pig was Previously Fed On, Initial Weight	9.14e+05
Independent	Guinea Pig	Triatomine Species, Life Stage, Number of Trials Guinea Pig was Previously Fed On, Initial Weight	9.22e+05

QIC: Quasi-likelihood Information Criterion

[a] The models were performed with a Triatomine Species*Life Stage interaction.

Supplemental materials related to the use of non-naive guinea pigs for triatomine feeding:

We wanted to see if the bloodmeal size would decrease over time as the guinea pigs would be repeatedly exposed to triatomine saliva and develop antibodies against salivary proteins. For each guinea pig, we recorded the number of trials the guinea pig was successfully fed on by an insect and used a GEE model to see if the size of bloodmeals would decrease over time as the guinea pigs' immune system was primed to the insects' salivary proteins [62,63]. Results and discussion are in the Supplemental Material (Figure S1; Table S16). All guinea pigs were considered naïve to triatomine salivary proteins at the beginning of the study. When a guinea pig was used and the insect did not feed, the bloodmeal size was recorded as 0 μL. In this model we also considered as covariates, the interaction between life stage (4th or 5th instar) and kissing bug species and the initial weight of the kissing bug including guinea pig as clustering variable. We also compared the QIC of independent and exchangeable models.

There was no difference in the size of insect bloodmeals that were taken on guinea pigs with different numbers of prior feeding events (P=0.57; Figure S1). Saliva serves as an important aid in the feeding process for triatomines as it has various anticlotting, anesthetic, antihistamine, and other properties [88]. Exposure to triatomine saliva can induce salivary antigens and other host-immune response [62,88], which could potentially decrease the amount of blood a triatomine can intake or affect the feeding process in future feeding events [89]. In our study, we did not see a significant difference of the bloodmeal size when the guinea pigs were repeatedly fed on by the triatomines.

Table S16: Parameter estimates of the best Gaussian generalized estimating equation model studying the blood volume ingested (μL) of triatomines

Parameter	Estimate (± S.E.)	*P*-value
Intercept—4th Instar, *R. prolixus*	4.05 (± 17.73)	0.820
T. gerstaeckeri	1.19 (± 7.80)	0.879
T. sanguisuga	5.05 (± 6.04)	0.404
Life Stage—5th Instar	22.49 (± 7.15)	0.0017*
Number of Trials Guinea Pig was Previously Fed On	2.25 (± 3.96)	0.570
Initial Weight (*g)	-274. 54 (± 116.33)	0.0183*
T. gerstaeckeri * 5th Instar	101.39 (± 25.49)	6.8e-05
T. sanguisuga * 5th Instar	5.09 (± 11.69)	0.663

*Statistically significant (P<0.05)

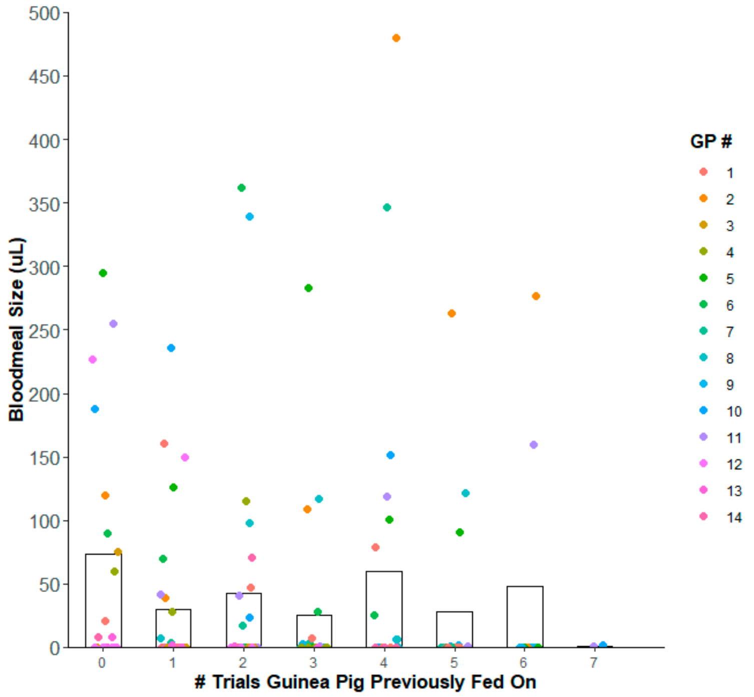

Figure S1: Bloodmeal sizes taken from guinea pigs by insects over time based on number of previous trials in which guinea pigs were fed on by triatomines. Data are only shown for the 59 insects that engaged in blood feeding during the trials. Each individual data point is a different insect that was used in the study with bars representing the mean bloodmeal size for each category. All guinea pigs were naïve to being fed on by triatomines at the beginning of the study (category zero). Each guinea pig was assigned a unique color.
